# Undiagnosed Cases of Human Pneumonia Following Exposure to *Chlamydia psittaci* from an Infected Rosella Parrot

**DOI:** 10.3390/pathogens10080968

**Published:** 2021-07-30

**Authors:** Anne-Lise Chaber, Martina Jelocnik, Lucy Woolford

**Affiliations:** 1 School of Animal and Veterinary Science, University of Adelaide, Roseworthy, SA 5371, Australia; lucy.woolford@adelaide.edu.au; 2 Genecology Research Centre, University of the Sunshine Coast, Sippy Downs, QLD 4557, Australia; mjelocni@usc.edu.au

**Keywords:** underdiagnosis, *Chlamydia psittaci*, psittacosis, zoonoses, wild birds, parrots, Australia

## Abstract

This report describes two cases of occupational exposure to *Chlamydia psittaci* following dissection of an infected Rosella (*Platycercus elegans*). The *C. psittaci* infections (with one of them resulting in diagnosed pneumonia and hospitalisation) were undiagnosed during routine medical investigations but later established due to epidemiological and clinical evidence, and molecular testing of the archived Rosella’ specimens. This case report stresses the importance of correct application and interpretation of diagnostic tests and the need to raise awareness about this zoonotic pathogen among medical practitioners and people exposed to potential animal carriers. Our findings suggest other infected individuals might be misdiagnosed and that *C. psittaci* (psittacosis) is likely to be underreported in Australia. This case highlights the need to operationalise the One Health concept. We call for improved communication between human and animal health service providers to allow accurate and rapid diagnosis of this zoonotic disease and raised awareness among medical practitioners. Further targeted surveys of wild birds (and other animals) should be conducted to improve assessment of risks to the general population and people working with or exposed to wild birds.

## 1. Introduction

*Chlamydia psittaci* is an obligate intracellular bacterium belonging to the family *Chlamydiaceae*. It is a well-known causative agent of psittacosis, an important occupational zoonotic disease of people working in close contact with birds (particular poultry), as well as chlamydiosis in birds and livestock [1].

In birds, such as poultry, pigeons, psittacines, and others, *C. psittaci* infections can lead to development of acute, subacute, and chronic forms of disease. In all forms, signs are non-specific but may include anorexia, diarrhoea, lethargy, weight loss, biliverdinuria, and ruffled feathers [2]. In some cases, mucopurulent or serous oculonasal discharge may be the only clinical sign [3]. Severe cases may result in dark green faeces, anorexia, dehydration, dyspnea, emaciation, and death [4]. The disease in humans can result in a range of clinical manifestations from asymptomatic infection or mild flu-like illness to systemic illness with severe atypical pneumonia. In symptomatic infection, abrupt onset of headache, fever, chills, malaise, and myalgia are typical, and may also develop a non-productive cough, breathing difficulty, and chest tightness [5,6]. Occupations considered at the highest risk for psittacosis include bird breeders, pet shop employees, and persons whose occupation places them at risk for exposure (e.g., employees in poultry slaughtering and processing plants, veterinarians, veterinary technicians, laboratory workers, workers in avian quarantine stations, taxidermists, farmers, wildlife rehabilitators, and zoo workers) [7,8,9]. Furthermore, home activities such as lawn mowing and gardening have also been associated with psittacosis during outbreaks [10]. *Chlamydia psittaci* is a genetically diverse species; some genotypes have been associated with a particular animal host, however with varying degree. Using the major outer membrane (*omp*A) gene marker, strains denoted genotypes A to F are most commonly associated with avian infections, and can also be detected in infected humans [1].

Australia is a home to a range of animal hosts. Amongst the wild bird species, highest prevalence of *C. psittaci* infections is noted in wild psittacines [11]. Despite several observations that wild psittacine birds could act as both direct and indirect reservoirs for human psittacosis and pose risk to public health, there are very few reports of psittacosis in Australia [11,12]. This paucity of reports is possibly reflecting lack of suspicion by medical practitioners or misinterpretation or incorrect application of diagnostic tests, not just in Australia but also globally [13,14]. 

Here, we describe two cases of occupational exposure to *C. psittaci* following dissection of an infected Adelaide Rosella (*Platycercus elegans*). The infections (with one of them resulting in pneumonia and hospitalisation) were undiagnosed during medical investigations but established due to epidemiological and clinical evidence and molecular testing of the archived Rosella specimens. 

## 2. Case Presentation

On the 18th of October 2019 (day one), Employee A, an ornithologist, was presented with an Adelaide Rosella (*P. elegans*) that had been found by Employee B (an office administrator) in the Adelaide hills region dazed and unable to fly the previous day. The bird was placed within a bird box for recovery but died overnight. The following day (day two), the bird was dissected on a bench in an open-air laboratory for routine tissue collection as per museum protocol, wearing gloves but no mask. The bird was found to be emaciated, and the liver was darker in appearance than normal with a green tinge suggesting cholestatic hepatopathy. Kidneys were bilaterally swollen suggesting nephrosis. Samples of liver and muscle were retained frozen (and kidney and liver samples retained in formalin) and the body of the bird was disposed as per protocol for biological waste material (off site medical incineration). 

On day nine (Oct 27) after contact with the Rosella, Employee A developed chills, fever, headache, diarrhoea, and some vomiting, with symptoms intensifying overnight. On day 11 (Oct 29), assessment by a general practitioner provided a tentative diagnosis of gastroenteritis, and no medication was prescribed. On day 12 (Oct 30), Employee A sought further confirmatory testing at a local hospital, where attending doctor tentatively diagnosed blood poisoning, and no medication was prescribed. On day 15 (Nov 2), after developing a slight dry cough, and constant fever and chills non-responsive to self-medication with paracetamol and ibuprofen, Employee A returned to a general practitioner who diagnosed dehydration and referred for hospitalisation and further testing. On admission to hospital, radiographs of the chest revealed pneumonia of the right lung. At this stage, no link was made to potential exposure to psittacosis, and it was not known at this stage that Employee B had also developed respiratory disease. 

Employee A was hospitalised between days 15 (Nov 2) and 22 (Nov 9), including two days in a Critical Care suite, and treated with oral antibiotics: ceftriaxone and azithromycin. Clinical signs began to abate three days after beginning antibiotic therapy. Serological testing using The SeroFIA™ Chlamydia immunofluorescent assays (MIF) kit (Savyon Diagnostics, Israel) and Liaison^®^ Mycoplasma pneumoniae IgG and IgM kit (DiaSorin Group Company, Ireland), performed on day 17 (Nov 4) revealed a *C. psittaci* IgG titre of 128 and *C. pneumoniae* titre of 128. *Mycoplasma pneumoniae* IgG testing by EIA was negative, but *Mycoplasma pneumoniae* IgM (EIA) was detected. On day 34 (Nov 21), repeated chest radiographs indicated resolution of pneumonia, and employee returned to work on day 38 (Nov 25). Repeated serological testing performed on day 60 (Dec 17) resulted in a *C*. *psittaci* IgG titre of 128, indicating no rise in antibodies 43 days after the first test was performed. Concurrent *C. pneumoniae* IgG titre was 512 but was considered a non-significant rise in convalescent antibodies as per the diagnostic laboratory report. Elsewhere, the fourfold titer increase is considered diagnostic of acute and current *C. pneumoniae* infection [15]. 

Employee B was also diagnosed with pneumonia (etiology undetermined, details of diagnostic testing performed not available), and was considered unfit for work for 20 days after showing respiratory symptoms. No further clinical information was available for Employee B. 

## 3. Follow Up Investigation in the Adelaide Rosella 

In January 2020, following consultation with veterinary disease specialists, frozen liver from the Adelaide Rosella was submitted to Gribbles Veterinary Pathology for nucleic acid detection of *C. psittaci* and returned a positive result. Thereby, we further wanted to determine genetic identity of the Adelaide Rosella strain using *C. psittaci*-specific multi-locus sequence typing (MLST) and *omp*A genotyping. A piece of frozen liver was placed in 70% ethanol and shipped to our laboratory for testing.

### 3.1. DNA Extraction and C. psittaci-Specific qPCR

The Adelaide Rosella liver segment, kept in 70% ethanol, was processed in 300 µL of sterile (Tris-EDTA) TE buffer with vortexing and heat lysis at 95 °C for 10 min, followed by DNA extraction using the QIAmp DNA blood and tissue kit (Qiagen, Australia) as per manufacturer instructions. DNA was eluted in 100 µl of AE buffer and stored at −20 °C until the qPCR analyses. The extracted DNA was checked for quality and concentration using Qubit^®^ 2.0 Fluorometer prior to qPCR assays. The extracted DNA (as is and in 1/10 dilution) was confirmed positive for *C. psittaci* using a species-specific real-time qPCR assays for *C. psittaci* [16] with the sample tested in duplicate, and a positive (*C. psittaci* isolate CR009 extracted gDNA) and negative (MilliQ water and mix only) control included in each assay. Testing and use of this liver sample was approved by the Animal Ethics Committee of University of the Sunshine Coast under tissue use exemption pathway (ANE2057). 

### 3.2. Molecular Characterisation 

*Chlamydia psittaci* genotyping was performed using the full length major outer membrane (*omp*A) gene sequencing and multi-locus sequence typing (MLST), targeting partial fragments of seven conserved housekeeping genes, as previously described [17]. Briefly, following the PCRs of MLST and *omp*A gene targets and Sanger sequencing (Macrogen, Korea), resulting sequences were confirmed for *C. psittaci* sequence type (ST) by interrogation against the online *Chlamydiales* MLST database (https://pubmlst.org/organisms/chlamydiales-spp, accessed on 25 May 2021) [18], and *omp*A genotype by BLASTn (https://blast.ncbi.nlm.nih.gov/Blast.cgi, accessed on 25 May 2021), respectively. The MLST and *omp*A sequences from this study were deposited in the *Chlamydiales* PubMLST database and Genbank (accession number MZ298912). Using the concatenated MLST sequences 3095 bp alignment of concatenated MLST sequences of the 37 global and Australian *C. psittaci* strains, we constructed a mid-point rooted approximately-maximum-likelihood phylogenetic tree, using FastTree 2.1.11 [19] as implemented in Geneious Prime 2021.1.1 (www.geneious.com, accessed on 25 May 2021).

## 4. Results

**Figure 1:**
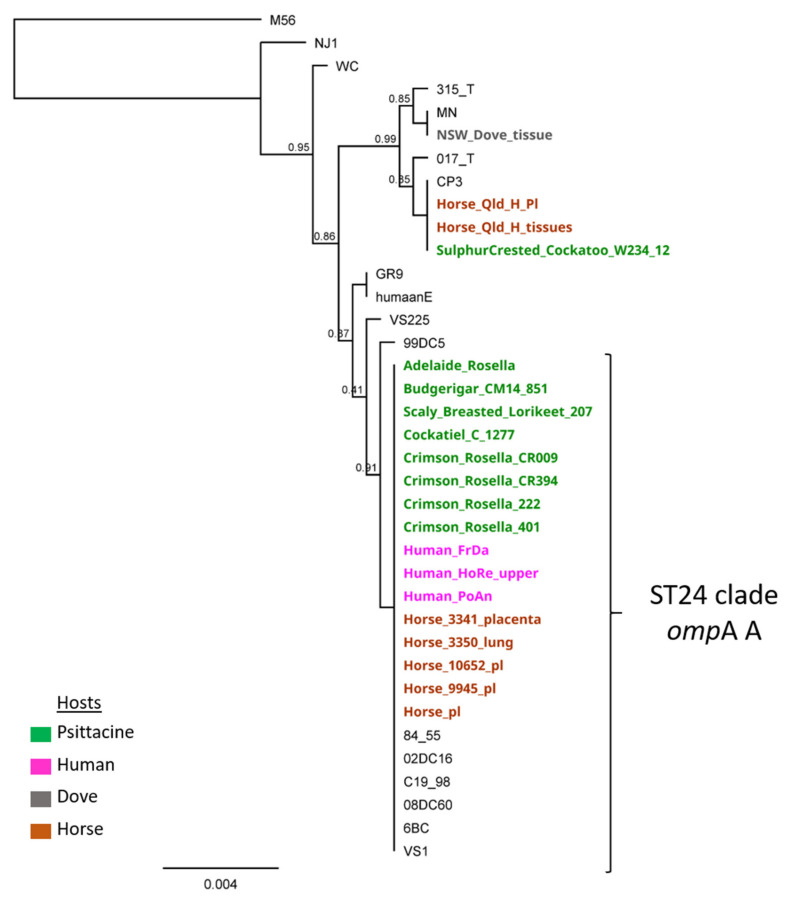
*Chlamydia psittaci* phylogenetic analyses including the Adelaide Rosella *C. psittaci* strain, associated with the zoonotic event described in this study. The mid-point rooted maximum-likelihood tree was reconstructed using the concatenated 3098 bp *C. psittaci* MLST genes alignment, and *C. psittaci* M56, an outgroup to root the tree. Bootstrap values are displayed on the nodes. Australian strains are displayed in bold, with their hosts denoted by different colours as outlined in the legend.

Molecular characterisation of the detected Adelaide Rosella *C. psittaci* DNA resolved ST24/*omp*A genotype A type strain. Phylogenetically, this strain is clustering in a well-supported ST24 clade with other Australian avian (psittacine), equine, and human strains, and globally distributed strains from a range of hosts (Figure 1). This strain type is commonly described in psittacine, equine, and human infections across Australia. 

## 5. Discussion 

Pets or wild psittacine birds are a major zoonotic reservoir of *C. psittaci*, with organisms excreted in the faeces and nasal discharge of clinically and sub-clinically infected birds [20]. As Australia is home to many wild bird species, direct as well as indirect contact with birds is well established and is considered a common risk factor for human psittacosis [21]. Most humans usually become infected after inhaling *C. psittaci* which has been aerosolised from dried faeces, feather dust, or respiratory secretions (e.g., sneezed droplets) of infected birds. However, other means of infection include handling of plumage and tissues of infected birds [2]. Post-mortem examination of infected birds and handling of infected cultures or eggs pose a particular human health risk [9,10,22]. Personal protective equipment (P2/N95 mask minimum) should be worn when handling infected or suspected infected birds or contaminated materials [23]. 

Onset of illness in humans follows an incubation period of 5–21 days [5], typically 10 days, but can be up to four weeks [6]. In light of the development of severe respiratory illness in two employees within 10 days after exposure to a clinically ill Adelaide Rosella, confirmed by nucleic acid detection to be infected by *C. psittaci*, psittacosis is the most probable cause of pneumonia in Employees A and B, despite inconclusive serological testing. Molecular characterisation of the Adelaide Rosella *C. psittaci* strain resolved ST24/*omp*A genotype A type strain, which is commonly found in Australian parrots, horses, humans, and widely associated with zoonotic events and pathogenicity [21]. In addition to confirmatory laboratory testing of the affected bird, post-mortem observations of emaciation, renomegaly, and hepatomegaly with green discolouration were consistent with avian chlamydiosis. Other pathological findings that may be observed in systemic avian chlamydiosis may include pericarditis, pulmonary congestion, fibrin exudation within body cavities, and splenomegaly with or without grey-white foci of discolouration [24]. 

Both confirmed and probable cases of psittacosis require notification to the Communicable Diseases Network Australia under the Series of National Guidelines (SoNG) for psittacosis. As per these guidelines, a *confirmed* case requires each of laboratory *definitive* evidence, clinical evidence, and epidemiological evidence. In the cases of the two employees reported here, both clinical and epidemiological evidence supported a diagnosis of psittacosis, however, laboratory definitive evidence was lacking. Laboratory definitive evidence for clinical confirmation requires a four-fold rise or greater in antibody titre against *Chlamydia psittaci* as demonstrated by micro immunofluorescence (MIF) on acute and convalescent sera (collected at least two weeks later) tested in parallel, or detection of *C. psittaci* by nucleic acid testing or culture [25]. Laboratory *suggestive* evidence for a probable case requires a single high total antibody level or detection of IgM antibody to *C. psittaci* by MIF, or a single high total antibody titre to *Chlamydia* species demonstrated by complement fixation (CF) in at least one sample obtained at least two weeks after onset of symptoms. 

In the case presented here, no significant rise in *C. psittaci* IgG antibody titre was observed between day 17 and day 60 post exposure. IgM MIF and CF testing was not performed. Another Australian study by Jones et al. where the same MIF test (The SeroFIA™ Chlamydia immunofluorescent assays) was used on 17 sera samples positive for *C. psittaci* IgG on the Chlamydia IgG recombinant enzyme linked immune assay (rELISA, Medac, Wedel, Germany) reported that no *C. psittaci*-specific antibodies were detected with MIF testing, six (35.3%) sera had detectable antibodies to other chlamydial species (such as *C. pneumoniae* or *C. trachomatis*), and the remaining eleven samples (64.7%) did not have specific antibodies to any chlamydial species [26]. A sputum sample was collected but not subjected to nucleic acid testing or culture and was discarded at the hospital a few days after collection, preventing any further investigation. Furthermore, a fourfold titre in *C. pneumoniae* IgG antibodies had been noted at Day 60. According to Kuo et al [15] and elsewhere, *C. pneumoniae* IgG titer of ≥ 512 is considered diagnostic for acute and current *C. pneumoniae* infection. In this case, it remains unclear why the fourfold *C. pneumoniae* titre rise was considered insignificant by the diagnostic laboratory report and not diagnostic for *C. pneumoniae*, whether this response was a result of a test cross-reactivity or true *C. pneumoniae* infection and/or co-infection with *C. psittaci*. As case definitions were not met for Employees A and B, the cause of pneumonia in these individuals remained unknown until confirmatory molecular testing was performed on the infected bird some months later. As a result, these two human cases of presumptive psittacosis were not reported to notifying bodies at the time of disease. 

The reasons for no observable increase in *C. psittaci* IgG antibody titre are unclear. At day 17, *Mycoplasma pneumoniae* IgG testing by EIA was negative, but *Mycoplasma pneumoniae* IgM (EIA) was detected, indicating either a false positive result or probable very early infection. The convalescent IgG titre was performed 60 days post exposure and 51 days after onset of clinical signs, outside of the recommended window of 10–30 days post onset of symptoms stipulated by the referring diagnostic laboratory protocols and by the Australian Public Health Laboratory Network [25]. A rise in convalescent IgG titre in the preceding weeks may therefore have been missed. Furthermore, patients infected with *Chlamydia psittaci* might remain seronegative, clinical decision-making concerning patient management based only on serology is unwise and should be avoided [27,28,29]. Real-time qPCR on BAL (bronchoalveolar lavage) or throat swab would have been appropriate to seek a diagnosis. A recent Australian study detected *C. psittaci* using qPCR methods in respiratory samples from cases where *Chlamydia* infections were not anticipated and/or suspected by the requesting clinician, further indicating that infections (such as *C. psittaci* in this study) are likely to be under-recognised causes of serious respiratory tract infections [30].

**Figure 2:**
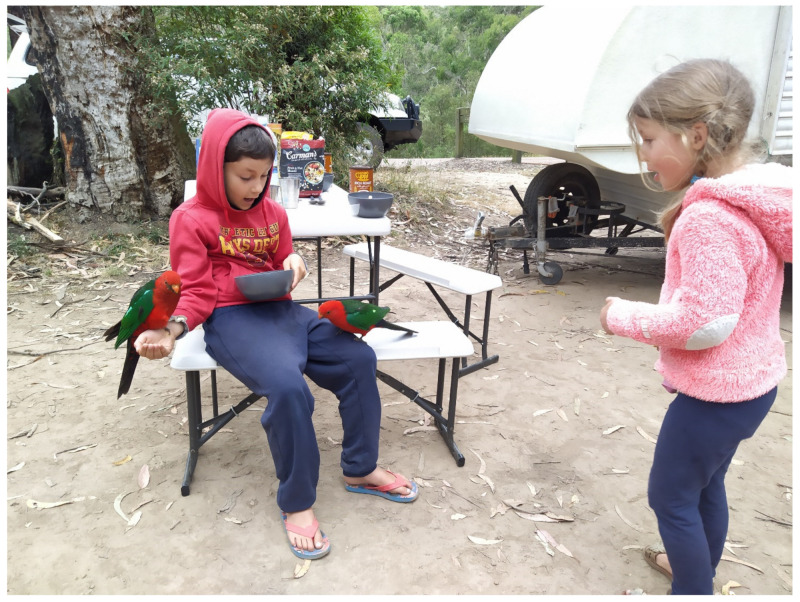
Picture featuring close contact between wild Australian king parrots (*Alisterus scapularis)* and a child posing a risk of zoonotic infection.

This case highlights the requirement for communication across all health sciences [5]. Adequate communication channels would ensure that any emerging challenges for public health can be actively addressed. Given the reported 9.8% subclinical carriage and 40% seroprevalence of *C. psittaci* in wild parrot populations from one distinct region of Australia [31], and that highly virulent and zoonotic *C. psittaci* strains belonging to the ST24/*omp*A genotype A clade have been genotyped from wild parrots across Australia [32], further targeted surveys of wild birds should be conducted to improve assessment of risks to the general population (Figure 2) and people working with wild birds, and raise awareness among medical practitioners. 

## 6. Conclusions

Despite both avian chlamydiosis and human psittacosis being long known and well characterised diseases, employee and employer knowledge of the risk of zoonotic exposure is not yet exhaustive nor widespread in at-risk industries. Increased awareness of the risks as well as broader education on the need/requirement for appropriate personal protective equipment (PPE) when handling or examining high risk animals is needed within the institution. Findings presented here also highlight that paired IgG serological testing alone is inadequate for the purpose of providing laboratory definitive evidence; detection of *C. psittaci* by nucleic acid testing or culture from sputum specimens or nasopharyngeal swabs or broncho alveolar lavage from the patient, and, ideally, laboratory testing of the source animal, should be encouraged to ensure greatest likelihood of correct diagnosis.

This case highlights the need to operationalise the One Health concept. The diagnosis and clinical management of those cases may have been improved if medical practitioners had a better understanding of this zoonosis, its diagnosis, and if they had considered including epidemiological inquiry and veterinary analysis in their medical investigation.

## Data Availability

The MLST and *omp*A sequences from this study were deposited in the *Chlamydiales* PubMLST database and Genbank. The Genbank accession number is: Adel_Rosella_ompA MZ298912.
